# Improving estimates of district HIV prevalence and burden in South Africa using small area estimation techniques

**DOI:** 10.1371/journal.pone.0212445

**Published:** 2019-02-22

**Authors:** Steve Gutreuter, Ehimario Igumbor, Njeri Wabiri, Mitesh Desai, Lizette Durand

**Affiliations:** 1 Division of Global HIV and TB, U.S. Centers for Disease Control and Prevention, Atlanta, Georgia, United States of America; 2 Division of Global HIV and TB, U.S. Centers for Disease Control and Prevention, Pretoria, Republic of South Africa; 3 School of Public Health, University of the Western Cape, Bellville, Cape Town, Republic of South Africa; 4 Division of Epidemiology and Strategic Information, Human Sciences Research Council, Pretoria, Republic of South Africa; University of Pretoria, SOUTH AFRICA

## Abstract

Many countries, including South Africa, have implemented population-based household surveys to estimate HIV prevalence and the burden of HIV infection. Most household HIV surveys are designed to provide reliable estimates down to only the first subnational geopolitical level which, in South Africa, is composed of nine provinces. However HIV prevalence estimates are needed down to at least the second subnational level in order to better target the delivery of HIV care, treatment and prevention services. The second subnational level in South Africa is composed of 52 districts. Achieving adequate precision at the second subnational level therefore requires either a substantial increase in survey sample size or use of model-based estimation capable of incorporating other pre-existing data. Our purpose is demonstration of the efficacy of relatively simple small-area estimation of HIV prevalence in the 52 districts of South Africa using data from the South African National HIV Prevalence, Incidence and Behavior Survey, 2012, district-level HIV prevalence estimates obtained from testing of pregnant women who attended antenatal care (ANC) clinics in 2012, and 2012 demographic data. The best-fitting model included only ANC prevalence and dependency ratio as out-of-survey predictors. Our key finding is that ANC prevalence was the superior auxiliary covariate, and provided substantially improved precision in many district-level estimates of HIV prevalence in the general population. Inclusion of a district-level spatial simultaneously autoregressive covariance structure did not result in improved estimation.

## Introduction

South Africa continues to have the highest burden of HIV in the world, with 7.1 million people living with HIV (PLHIV), an estimated 270,000 new HIV infections, and 110,000 AIDS-related deaths in 2016 [1]. Nationally, HIV prevalence among 15-49 year-olds living in South Africa was estimated to be 18.8% in 2012 but—importantly—varied at the provincial level from 7.8% in the Western Cape to 27.9% in KwaZulu-Natal [2].

Allocation of resources for HIV prevention and treatment to areas of greatest need is key to epidemic control and saving lives. For example, geographic prioritization of combination prevention resources has the potential to avert substantially more HIV infections than does uniform distribution of those same resources [3]. The current approach to reaching the ambitious HIV prevention, care and treatment goals relies on targeting key sub-national locations and populations to end the HIV/AIDS epidemic by 2030 [4, 5]. South Africa’s new “focus for impact” strategy concentrates efforts in the 27 districts most affected by HIV [6]. Those and similar public health responses must rely on more granular epidemiological and programmatic information describing local epidemics [5].

However, existing data sources do not usually provide reliable sub-national estimates of HIV prevalence [7]. For example, monitoring of HIV prevalence in countries has historically relied on surveillance among pregnant women included in semi-systematic convenience samples from (sentinel) antenatal care (ANC) clinics [8]. However, there are many important biases inherent in ANC sentinel surveillance [9], and those data require careful analysis and interpretation [10]. Pregnant women who attend sentinel ANC clinics may not represent the broader population in terms of HIV burden or transmission dynamics [8, 9]. Urban populations may be over-represented among ANC sites [11]. Pregnant women are, by definition, sexually active and therefore may be having unprotected vaginal sex at a greater rate than the general population of women.

To augment available strategic information on HIV prevalence, and to address some of the identified shortcomings in the ANC surveillance data in South Africa, national probability-based household HIV prevalence surveys were introduced in 2002, and were repeated during 2005, 2008, 2012 [2] and 2017. Similar Demographic and Health Surveys (DHS) have incorporated HIV testing in many other countries [12]. Probability-based health surveys employ complex multi-stage cluster sampling with linked anonymous HIV testing to obtain representative samples from the population of persons who consent to testing.

Sample sizes for the South African surveys are calculated to detect a 5% change in HIV prevalence in each of the five reporting domains (sex, age, race, locality type and province). Sample sizes also provide a 4% margin of error in the prevalence estimates from the reporting domains, including the domain of the nine provinces of South Africa [2]. However, the 2002, 2005, 2008 and 2012 surveys do not provide adequate precision for estimation of prevalence within the 52 districts which comprise the nine provinces, and the 2017 survey prioritized only certain districts for HIV prevalence estimation. Achieving adequate universal district-level precision from a probability survey would require substantial increases in sample size. Therefore, model-based methods which incorporate multiple sources of data are needed which can improve precision of district level estimates without requiring large increases in sample size.

To date, subnational estimation of HIV prevalence from probability surveys has been based upon several approaches. PrevR [13] uses kernel density estimation to interpolate from geographic point estimates of HIV prevalence from a DHS to produce continuously scaled choropleth maps. PrevR does not incorporate the uncertainty of estimation from the DHS, and cannot produce quantitative estimates of uncertainty. Bayesian kriging of individual-level data from a household survey has been used for subnational estimation and display of HIV prevalence in South Africa [14]. Fully Bayesian geostatistical models have been used to estimate subnational HIV prevalence from individual-level health survey data [15–17]. Unlike PrevR, those Bayesian models accommodate the uncertainty in the data and can produce probability intervals on the estimates. However, all of those approaches used only data from a probability survey, and therefore precision remained limited largely by the information content of the survey.

Unit- and area-level small-area estimation (SAE) methods [18–20] enable augmentation of survey data with, for example, data from convenience sampling or routine monitoring to improve precision over design-based domain estimation using the survey data alone. Unit-level SAE models require that auxiliary data are available from each primary sampling unit present in the survey data, which limits the choice of auxiliary data. Area-level models require only that auxiliary data are available for the estimation domains from the survey. Suppose, for example, that estimates are required for the second subnational (SNU2) level, and data are available from a probability survey designed to produce estimates at the first subnational (SNU1) level. Imprecise design-based domain estimates at the SNU2 level are computed using conventional survey methods. The key idea is to model those health survey-based prevalence estimates as functions of predictors from data which are external to the survey. The precision of the resulting small-area estimates is a function of the precision of the direct domain estimates and the degree to which the auxiliary covariates predict those domain estimates. For example, area-level synthetic SAE of HIV prevalence in local municipal areas of South Africa was based on data from a national household survey and five demographic variables [21].

Despite well-known relations between HIV prevalence in the general population and prevalence among pregnant women who attend ANC clinics [4], to our knowledge, no previous subnational estimation efforts have exploited the information content of the latter to improve estimation of the former. We postulated that area-level SAE of HIV prevalence for the 52 districts of South Africa would produce substantially more precise estimates than conventional design-based domain estimation because auxiliary estimates of HIV prevalence from ANC surveillance should be good predictors of prevalence in the general population. Our aim was to demonstrate the potential for improved district-level estimation using 2012 HIV household survey domain estimates by incorporating HIV prevalence among pregnant women attending ANC clinics and other covariates.

## Materials and methods

### Data

We used data from the 2012 South African National HIV Prevalence, Incidence and Behavior Survey (SABSSM IV) [2]. That survey sampled residents from 15 randomly selected households within each of 1,000 census enumeration areas (EAs) which were selected randomly with probability proportional to size from the 86,000 EAs of South Africa. A total of 42,950 individuals were contacted, 38,431(89.5%) consenting persons were interviewed and 28,997(67.5%) blood specimens were tested for HIV. We computed conventional Horvitz-Thompson design-based domain estimates [22] of district-level HIV prevalence (proportion of HIV-positives) among persons of all ages and associated standard errors from those data, henceforth called direct estimates. District-level auxiliary predictors were: HIV prevalence among women making the first visit of their pregnancy to public ANC clinics participating in the 2012 National Antenatal Sentinel HIV & Herpes Simplex Type 2 Prevalence Survey in South Africa (ANCSS) [23]; population density (km^-2^); the percentages of housing units which were “formal” dwellings [24]; dependency ratio (ratio of the numbers of residents aged 15-64 years to those younger than 15 years and older than 64 years) [24]; socio-economic quintile (1-5) [25]; and maternal mortality rate (maternal deaths within six weeks of parturition per 10,000 pregnancies) [25]. For the ANCSS, HIV testing was performed from 34,260 women between the ages of 15-49 during their first visits to the 1,497 ANC sites which had been selected randomly with probability proportional to size for inclusion in the 2012 round of surveillance. The auxiliary predictors were chosen based on both a priori assumptions about potential correlations with HIV prevalence and their availability. We do not assume that the set of auxiliary predictors selected for this demonstration provide the best possible predictions of HIV prevalence in the general population. SAE requires only that some among them contain information about HIV prevalence in the general population.

### Ethics statement

The SABSSM IV survey protocol was approved by the Research Ethics Committee of the South Africa Human Sciences Research Council as well as by the Associate Director of Science of the National Center for HIV and AIDS, Viral Hepatitis, STD and TB Prevention at the US Centers for Disease Control and Prevention (CDC). All persons who agreed to participate in the SABSSM IV survey were required to provide either written or verbal consent for both the interview and dry blood-spots specimen collection. Verbal consent was applied where the respondent was illiterate. Parents and guardians of children under 18 years of age were asked to give informed consent for inclusion of their children in the SABSSM IV survey as well as for providing a blood specimen for HIV testing. Children aged 7 to 11 years were required to confirm their assent by placing a tick or cross in a demarcated box in addition to providing written assent by means of a signature (where possible). Those aged 12 to 17 years were required to provide written assent by means of a signature. The identities of survey participants were not disclosed to the authors.

Participation in the 2012 ANCSS was voluntary and with informed consent [23]. For reasons of confidentiality, testing was done on anonymous unlinked samples. A unique bar code was assigned to each participant, and was used to link the demographic and socio-economic variables with the laboratory results while maintaining anonymity of the survey participant. The present study used published district-level summaries which contained no identifying information about ANCSS participants.

This study was performed under the terms of the CDC protocol titled “Small Area Estimation of HIV Prevalence: A protocol for analysis of publicly available census, health survey and surveillance data for improving district-level estimation of HIV prevalence in South Africa”, which was approved by both the CDC and the South Africa Human Sciences Research Council. This study does not constitute human subjects research, as the primary intent is public health practice.

### Statistical models

We used variations of the basic area-level model [19, 26] to estimate district-level HIV prevalence in the general population. Briefly, the basic area-level model combines survey-based direct area-level domain estimates, auxiliary (out-of-survey) predictors, and area-specific random effects which borrow strength across areas. Conventionally, the area-level random effects are assumed to be independently normally distributed, but spatial covariance structures can also be modeled. We fitted 12 variations of the basic area-level model, which differed in the inclusion of auxiliary predictors and assumptions about the random effects. We fitted models which assumed independently normally distributed random effects and, alternatively, simultaneously autoregressive (SAR) random effects [27] based upon spatial adjacency of the districts of South Africa. The S1 Appendix provides additional details of our models. SAE was performed using the sae package [28] for R [29]. Code and data are provided in the S1 Data and Code.

The response variable for all models was the logit transformation of the direct domain estimates of HIV prevalence proportions among all persons, regardless of age or sex, and sampling error variance was estimated as Delta-method approximation using the variances of the domain estimates. Eleven of the 12 models included the logit transformation of district-level ANC prevalence proportion as an auxiliary predictor. Model 1 included only the logit of ANC prevalence proportion. Models 2–5 augmented model 1 with inclusion of the district-level percentages of formal dwellings, dependency ratio, socio-economic quintile and maternal mortality rate, respectively. Model 6 augmented model 2 with inclusion of the dependency ratio. Model 7 augmented Model 6 inclusion of maternal mortality rate. Model 8 augmented model 7 with inclusion of the percentages of births which occurred in health care facilities. Model 9 was reduced from model 8 by deletion of the logit of ANC prevalence, and provides the contrast needed to assess the value of ANC prevalence. Models 10–12 relax the assumption of independent model errors in models 1, 2 and 8, respectively, with inclusion of a SAR spatial covariance structure. Relative model performance was assessed using the Akaike Information Criterion (AIC) [30]. AIC balances model fit against model complexity; smaller values of AIC indicate relatively better predictive ability. AIC is a dimensionless relative measure, and differences of 5 between models are customarily considered to be important.

District-level estimates of the burden of HIV infection, defined as the number of PLHIV, were obtained as the product of district-level HIV prevalence from the best fitting model and district population size obtained from Statistics South Africa [24].

## Results

The correlation coefficient between the district-level survey domain estimates of HIV prevalence in the general population and HIV prevalence among pregnant women was 0.71 (S1 Table), so that the latter explains approximately 50% of the variation in the former. In contrast, the correlation coefficients between district-level prevalence in the general population and the percentage of formal dwellings among residences, dependency ratio, socio-economic quintile, maternal mortality rate, percentages of births in health facilities and population density were –0.36, 0.29, -0.28, –0.14, 0.09 and 0.01, respectively. Thus, we should expect HIV prevalence among pregnant women to make the largest contribution of information in SAE.

The AIC-best model (model 3) included only ANC prevalence and dependency ratio as out-of-survey predictors (Table 1). However, pairwise AIC differences among models 1–3, 5–7, 10 and 11 were smaller than 5, and therefore all of those models are plausible alternatives. Differences among log-likelihoods were small except for differences between either models 9 or 12 and the others, so that AIC differences are generally dominated by model complexity rather than fit. ANC prevalence was the superior predictor of survey-based prevalence. Inclusion of auxiliary predictors other than dependency ratio (model 3), resulted in larger AIC values than ANC prevalence alone (model 1). Further, exclusion of ANC prevalence yielded the worst of the models (model 9). Inclusion of the SAR spatial effect of district location did not improve model fit (i.e., compare models 1 with 10, 3 with 11 and 8 with 12).

**Table 1:** Model comparison based on the log-likelihood and Akaike Information Criterion (AIC; smaller is better). P_ANC_ denotes the HIV prevalence proportion among pregnant women obtained from surveillance at antenatal care (ANC) clinics. Other auxiliary predictors are: Formal = formal dwellings (%); DR = dependency ratio; SEQ = socio-economic quintile; MMR = maternal mortality rate; HFB = births in health-care facilities (%). Covariance structures (Cov) are independence (Ind) or simultaneously autoregressive (SAR).

Model	Predictor(s)	Cov	Log-likelihood	AIC
1	logit(P_ANC_)	Ind	−23.96	53.92
2	logit(P_ANC_) + Formal	Ind	−23.42	54.85
3	logit(P_ANC_) + DR	Ind	−22.37	52.73
4	logit(P_ANC_) + SEQ	Ind	−22.84	59.68
5	logit(P_ANC_) + MMR	Ind	−23.75	55.51
6	logit(P_ANC_) + Formal + DR	Ind	−22.36	54.73
7	logit(P_ANC_) + Formal + DR + MMR	Ind	−22.34	56.67
8	logit(P_ANC_) + Formal + DR + MMR + HFB	Ind	−22.20	58.39
9	Formal + DR + MMR + HFB	Ind	−37.81	87.63
10	logit(P_ANC_)	SAR	−23.95	55.91
11	logit(P_ANC_) + DR	SAR	22.37	54.73
12	logit(P_ANC_) + Formal + DR + MMR + HFB	SAR	−31.80	77.61

**Fig 1:**
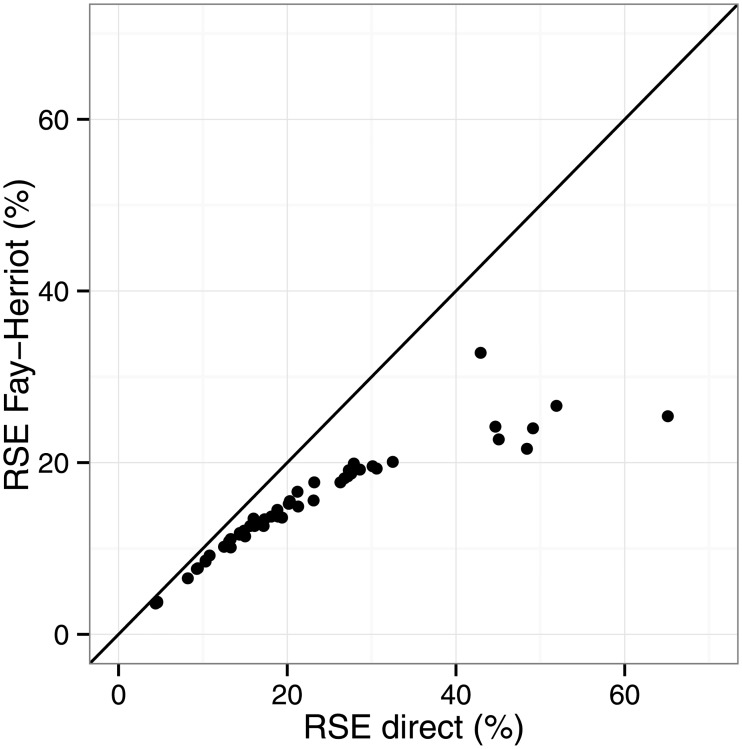
Relative standard errors of district-level estimates. Relative standard errors (RSE) of the 52 South African district-level HIV prevalence estimates obtained from the AIC-best Fay-Herriot area-level model and direct (survey domain) estimation.

**Fig 2:**
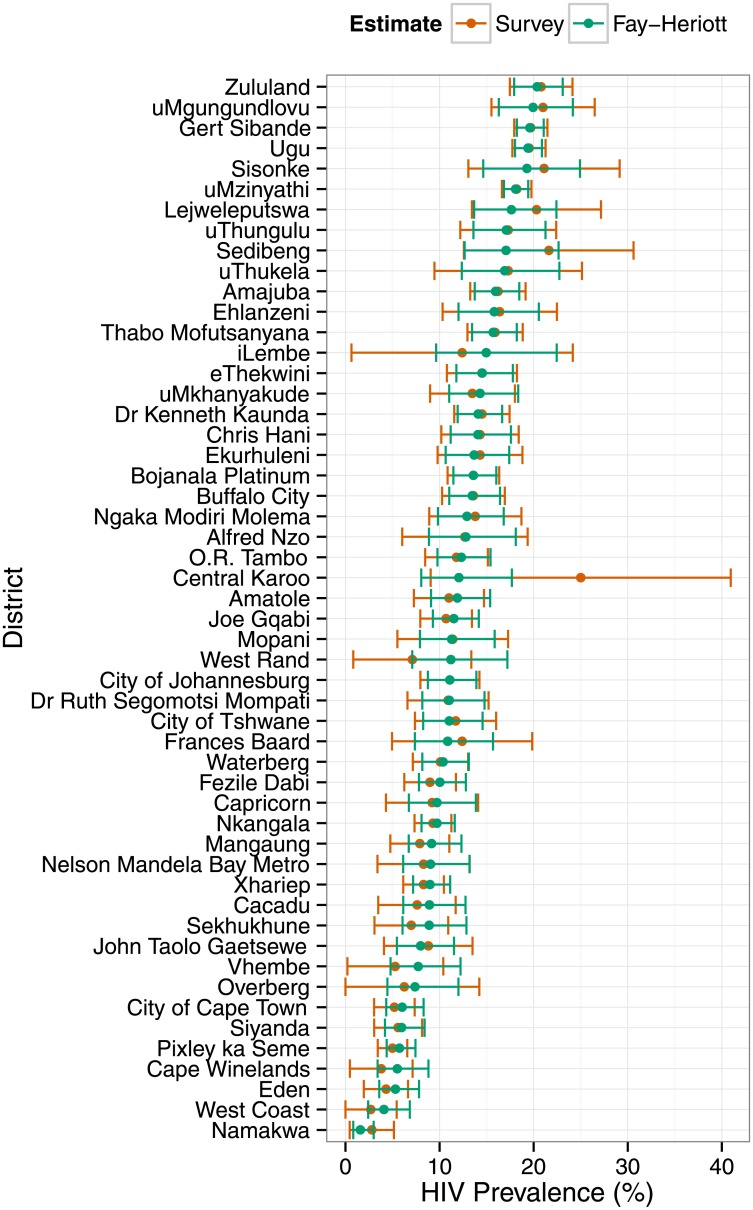
HIV prevalence in the districts of South Africa, 2012. District-level estimates of HIV prevalence and 95% confidence intervals from the 2012 South African National HIV survey, and from Fay-Herriot small-area estimation. Numeric values are provided in S2 Table.

The AIC-best Fay-Heriott model (model 3) produced district-level estimates of HIV prevalence which were typically more precise, and never less precise, than the direct survey-based domain estimates (Fig 1). The reduction in relative standard errors of estimation (RSE) were greatest among districts which produced the least precise direct estimates; SAE gains little where direct estimates are precise. Assuming, for example, that “useful” estimates are those for which RSE ≤ 20%, then model 3 produced useful estimates from 15 of the 23 districts for which direct estimation failed to produce useful estimates. Accordingly, the Fay-Herriot estimates of HIV prevalence had narrower 95% confidence intervals than the direct estimates (Fig 2). Additionally, some point estimates of HIV prevalence differed rather substantially (e.g., Sedibeng, iLembe, Central Karoo and West Rand). The design-based survey domain estimate of HIV prevalence in West Rand was of little value for lack of precision, and likely misleading, whereas the small-area estimate was similar to surrounding districts and consistent with programmatic data.

**Fig 3:**
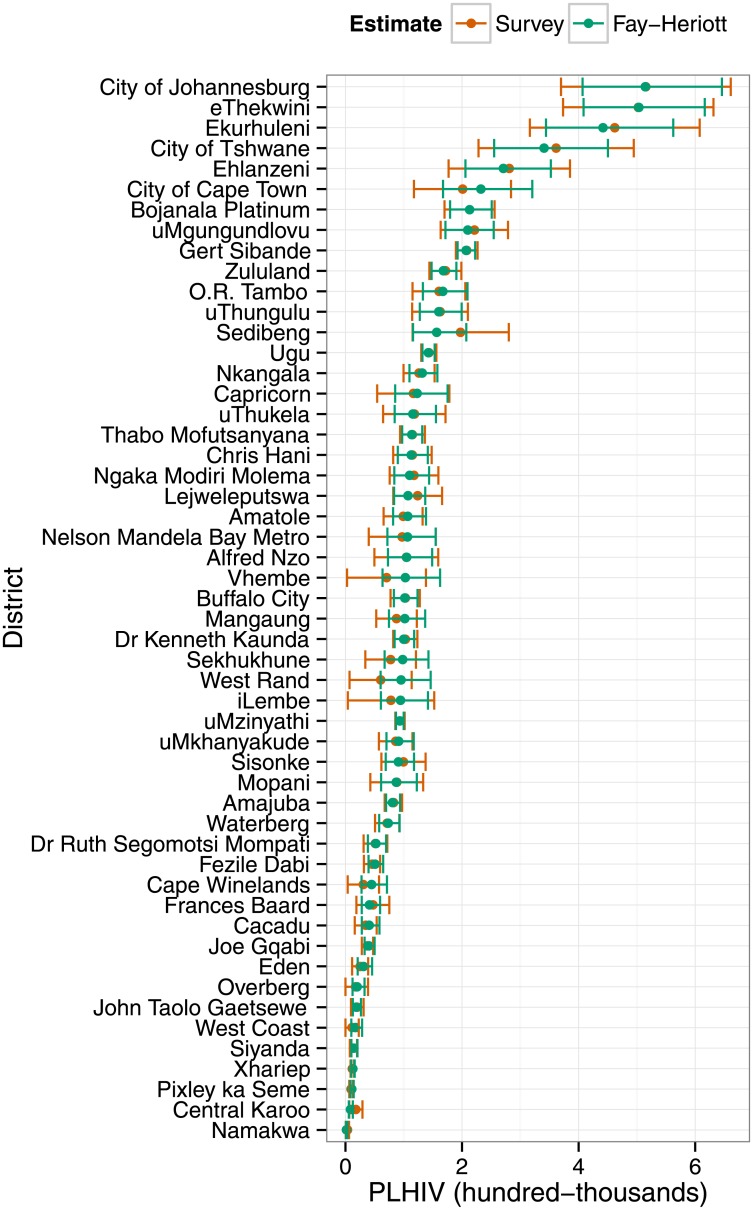
HIV burden in the districts of South Africa, 2012. District-level estimates of numbers of people living with HIV (PLHIV) and 95% confidence intervals from the 2012 South African National HIV Survey and from Fay-Herriot small-area estimation.

HIV prevalence is easily translated to the numbers of PLHIV. Again, confidence intervals for Fay-Herriot small-area estimates of numbers of PLHIV were generally narrower (and never wider) than direct survey-based estimates (Fig 3). Rankings based upon point estimates also differed slightly, but less so than the prevalence estimates because of the large effect of district population size on numbers of PLHIV.

**Fig 4:**
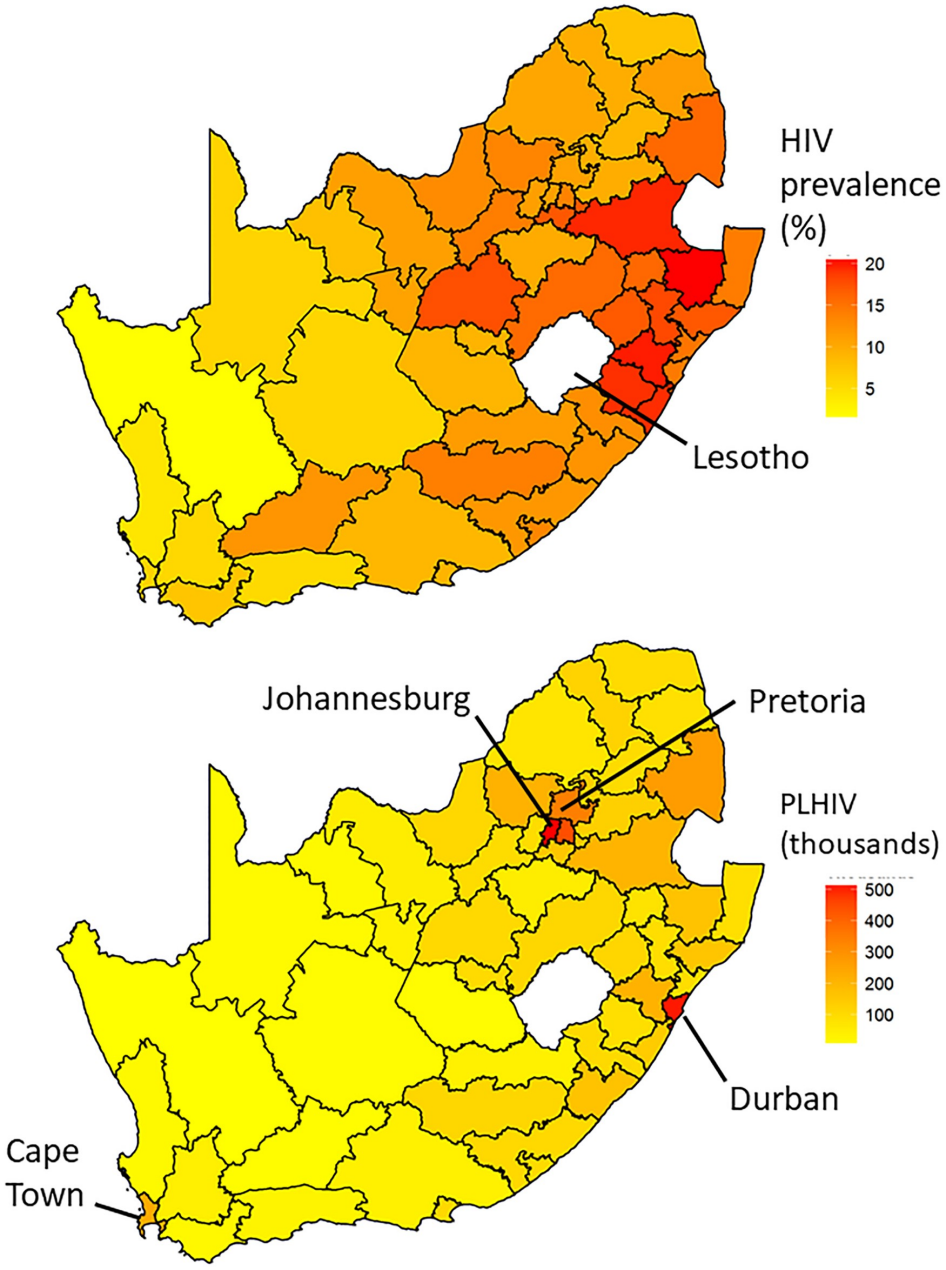
Geographic distribution of HIV prevalence and burden. Geographic distribution of HIV prevalence (top) and numbers of people living with HIV (PLHIV, bottom) in South Africa, 2012, based upon Fay-Herriot small-area estimation. Numeric values of estimates are provided in S2 Table.

A strong east-west gradient in HIV prevalence is evident (Fig 4, upper). However, the population is concentrated in eastern urban centers (City of Johannesburg and Tshwane [Pretoria] in the northeast, and eThekwini [Durban] on the east coast). Therefore the numbers of PLHIV are concentrated in those urban centers, along the eastern borders with eSwatini (formerly Swaziland) and Mozambique, and to a lesser extent around the City of Cape Town on the southwest coast (Fig 4, lower).

## Discussion

Successful HIV epidemic control in the era of increasingly limited resources will require targeted delivery of the “right things, in the right place and at the right time” [5]. Data-driven public health actions that can deliver on this goal will require valid and reliable estimates of HIV prevalence and numbers of PLHIV in areas containing small populations, including those which are inadequately sampled by health surveys. Estimates should be as precise as possible to minimize the risk of inefficient allocation of HIV program services. Multiple, statistically principled, methods are available to estimate quantities over small geographic areas [16, 17, 19, 31, 32], which differ in data requirements and computational ease. The Fay-Herriot model provides epidemiologists and public health planners with an effective and easily implemented approach to estimate HIV prevalence and numbers of PLHIV in small subnational areas.

The improvement in precision that we observed over survey domain estimation was largely due to the availability of published district-level estimates of HIV prevalence among pregnant women obtained from ANC surveillance. Data on HIV prevalence among pregnant women, either from sentinel surveillance or routine reporting from prevention of mother-to-child transmission of HIV services, are available from most sub-Saharan African nations [33]. Therefore the feasibility of area-level SAE of HIV prevalence in the general population depends largely on the availability of probability-survey data including HIV testing. Outside of South Africa, the DHS-Plus [34] and Population-based HIV Impact Assessments (PHIAs) [35] provide those data from many sub-Saharan nations. The PHIA surveys include testing for major metabolites of anti-retroviral medications and estimation of serum HIV loads and, in principle, enable SAE of the entire 90-90-90 cascade (proportions of HIV-positive people who know their status, on treatment, on treatment and virally suppressed).

Inclusion of the spatial SAR covariance structure did not improve the small-area estimates of HIV prevalence in our data and models. However, that result does not imply that spatial correlation is unimportant in other contexts. The SAR covariance structure is coarse-grained in area-level models. First, it is based on binary indicators of whether pairs of districts are adjacent. In contrast, distance between districts is also likely to matter. Second, the spatial grain (districts) is too coarse to capture finer scale correlations which may exist, say among census enumeration areas. For example, spatial correlation is extremely important in geostatistical models which incorporate more sensitive models for spatial structure [16, 17].

Small area estimation can improve estimates of HIV prevalence in small, unplanned analysis domains. However, precision is ultimately limited by survey sample size. Therefore there is no guarantee that any method for estimation in small unplanned domains will yield desirable precision in any domain. The best that can be done is to use the best-available data and then critically evaluate the utility of the gains for each small domain.

Policy makers can use these second-level subnational estimates to more efficiently reallocate resources. Allocation of resources to areas with high HIV prevalence and large numbers of PLHIV can provide care and treatment to more people, while saving money. Additionally, programs can use those estimates to better understand the epidemic and plan where to place intervention services.

The general east-west gradient in HIV prevalence observed in our analysis is consistent with the heterogeneous distribution of HIV within South Africa as described by others. A model-based evaluation of factors accounting for this spatial patterning of HIV in South Africa highlighted the role of differing prevalence of male circumcision and the frequency of non-marital sexual activity [36]. It is likely that these, and sundry other cultural, demographic, behavioral and individual HIV risk factors are indeed important epidemic drivers of this prevalence pattern in South Africa.

We chose the Fay-Herriot model because it is simplest method for SAE from aggregated area-level predictor covariates, and is easily implemented using freely available software. Data which are aggregated to subnational levels are often readily available because frequently public health programs managed and census results are reported at those levels. Estimation is straight-forward once the data have been obtained. The basic Fay-Herriot area-level model requires the assumption that area-level level variances in the response variable are known. Uncertainty may be underestimated as a result, and future applications of SAE of HIV prevalence, numbers of PLHIV and HIV incidence may benefit from the application of more sophisticated methods which relax that assumption [37].

## Conclusion

The basic area-level “Fay-Herriot” model is a viable choice for estimation of HIV prevalence and numbers of PLHIV in small, unplanned survey domains. Major advantages include use of commonly available aggregated area-level covariates, and relative ease of implementation using freely available software. We assert that the basic area-level model is the most easily implemented alternative, and requires only very modest computational resources. Our results demonstrate that incorporation of data from ANC clinics, commonly available from sub-Saharan Africa, can yield better estimates of HIV prevalence in the general populations of small areas than the types of behavioral demographic variables which have been incorporated previously [16, 21]. Our small-area estimates of HIV prevalence in the general population of the districts of South Africa were often more precise—and never less precise—than the design-based domain estimates upon which they are based.

## Supporting information

S1 Appendix Model description. A PDF file containing a detailed description of the basic area-level model applied to estimation of HIV prevalence.
(PDF)

S1 Data and Code Data and R code. A ZIP file containing district-level data and R code needed to reproduce our results.
(ZIP)

S1 Table Correlations among variables. A PDF file containing a table of correlations among variables.
(PDF)

S2 Table Estimates. A spreadsheet containing district-level estimates and their 95% confidence intervals.
(XLSX)
