# Supplementary material for: Improving estimates of district HIV prevalence and burden in South Africa using small area estimation techniques
Source: PLoS One. 2019 Feb 22;14(2):e0212445. doi: 10.1371/journal.pone.0212445 (PMC6386240; doi:10.1371/journal.pone.0212445)
Supplement: S1 Appendix — A PDF file containing a detailed description of the basic area-level model applied to estimation of HIV prevalence. (PDF) [file pone.0212445.s001.pdf]

Model Description

Improving estimates of district HIV prevalence and burden in South Africa using small area estimation techniques

Let $\bar{y}_i$ denote the conventional design-based (direct) domain estimate of the mean some quantity from small area $i, i = 1, \ldots, m$, and let σ_i^2 denote the associated variance of $\bar{y}_i$. Under design-based sampling theory,

$$\bar{y}_i = \theta_i + \epsilon_i$$

where θ_i is the true but unknown quantity estimated by $\bar{y}_i$, and ϵ_i is random error having mean 0 and variance σ_i^2. Under the basic area-level model [1,2], we further assume that the ϵ_i are normally distributed. Suppose we have some number h covariates from auxiliary data. Then we assume that each θ_i is a linear function of the auxiliary covariate vector $x_i = [x_{1,i}, \ldots, x_{h,i}]'$ given by

$$\theta_i = x_i\beta + \nu_i$$

where β is a regression parameter vector of length $h+1$ and the ν_i are normally distributed random errors, independent of the ϵ_i, and having mean 0 and variance σ_ν^2. Combining those equations gives

$$\bar{y}_i = x_i\beta + \nu_i + \epsilon_i$$

which is a mixed-effects linear regression model. The parameters β and σ_ν^2 are estimated conditional on the σ_i^2, which are provided as data. Typically, the σ_i^2 are replaced by S_i^2, the direct estimate of sampling variance obtained from the survey, but Bayesian extensions enable modeling the σ_i^2 [3]. The "Fay-Herriot" small-area estimates $\hat{y}_i$ of θ_i are given by the James-Stein shrinkage estimator

$$\hat{y}_i = \gamma_i\bar{y}_i + (1 - \gamma_i)\, x_i\hat{\beta}$$

where

$$\gamma_i = \frac{\hat{\sigma}_\nu^2}{\hat{\sigma}_\nu^2 + \sigma_i^2}$$

is the ratio of the model error variance to the total variance.

Note that each $\hat{y}_i$ is a weighted sum of the corresponding direct estimate $\hat{y}_i$ and the regression (synthetic) estimate $x_i\hat{\beta}$, and $\hat{y}_i$ is shrunk from the domain estimate $\bar{y}_i$ toward the synthetic estimate. The Fay-Herriot estimate

$\hat{y}_i$ is near $\bar{y}_i$ for values of values of $\hat{\sigma}_\nu^2$ that are large relative to σ_i^2, and near $x_i\hat{\beta}$ where $\hat{\sigma}_\nu^2$ is small relative to σ_i^2. That is, the direct domain estimates will dominate when their precision is high, and the synthetic estimates will dominate where the precision of the direct estimates is low relative to the model error variance. Equivalently, the area-level model will perform best where covariates are available which are strongly correlated with the direct domain estimates. By definition such covariates—originating from outside the survey—provide additional information about the unknown θ_i.

The assumption of independently distributed model errors ν_i can be relaxed by incorporating simultaneously autoregressive (SAR) spatial covariance structure [4], for which we assume $\nu_i \sim N(\mathbf{0}, \mathbf{\Sigma})$, where $\mathbf{\Sigma}$ is the $m \times m$ covariance matrix given by

$$\mathbf{\Sigma} = \sigma_\nu^2 \left[(\mathbf{I} - \rho\mathbf{W}) \left(\mathbf{I} - \rho\mathbf{W}^T\right) \right]^{-1}$$

where $\mathbf{I}$ is the $m \times m$ identity matrix, $\mathbf{W}$ is an $m \times m$ row-standardized matrix having off-diagonal elements $(i, j), i \neq j$ equal to $1/k_i$ if district j is adjacent to district i, where k_i is the number of districts adjacent to i, and 0 otherwise, and ρ is the spatial autocorrelation parameter, which is estimated from the data.

Note that the SAR covariance structure is rather crude in that it is implemented at the area level, whereas stronger spatial correlations might exist at smaller spatial scales, and because it is based only upon whether or not pairs of areas are adjacent to each other rather than the distances between pairs of areas. For that reason, the SAR covariance structure is likely to provide useful information only where informative covariates are unavailable.

For our example, we set $y_i = \text{logit}(p_i)$ and $x_{1i} = \text{logit}(p_{ANCi})$ where the p_i are the direct domain estimates of district-level HIV prevalence proportions from the survey, and the p_{ANCi} are the prevalence proportions among pregnant women who obtained antenatal care services from clinics in district i. The logit transformation maps prevalence proportions to the real line and aids the normality assumption about the ν_i. We also considered other covariates as described in the text. The variance of $y_i = \text{logit}(p)$ was approximated by the Delta method and is given by

$$\text{Var}(\hat{y}_i) \cong \left(\frac{\theta_i}{1 - \theta_i}\right)^2 \text{Var}(p_i).$$

where the $\text{Var}(p_i)$ are the variance estimates for the p_i.

Model fitting was performed using the sae package [5] for R [6].

References

1. Fay RE, Herriot RA. Estimates of income for small places: An application of James-Stein procedures to census data. Journal of the American Statistical Association. 1979;74(366):269–277.

2. Rao JNK, Molina I. Small Area Estimation. 2nd ed. Wiley Series in Survey Methodology. New York: Wiley; 2015.

3. You Y, Chapman B. Small area estimation using area level models and estimated sampling variances. Survey Methodology. 2006;32(1):97–103.

4. Marhuenda Y, Molina I, Morales D. Small area estimation with spatio-temporal Fay-Herriot models. Computational Statistics and Data Analysis. 2013;58:308–325. doi:10.1016/j.csda.2012.09.002.

5. Molina I, Marhuenda Y. sae: An R Package for Small Area Estimation. The R Journal. 2015;7(1):81–98.

6. R Core Team. R: A Language and Environment for Statistical Computing; 2018. Available from: http://www.R-project.org/.
