# Supplementary material for: Improving estimates of district HIV prevalence and burden in South Africa using small area estimation techniques
Source: PLoS One. 2019 Feb 22;14(2):e0212445. doi: 10.1371/journal.pone.0212445 (PMC6386240; doi:10.1371/journal.pone.0212445)
Supplement: S1 Table — A PDF file containing a table of correlations among variables. (PDF) [file pone.0212445.s003.pdf]

S3 Table. Correlations among variables. HIV prevalence among pregnant women was the strongest predictor of HIV prevalence in the general population, followed by the percentage of residence which were "formal" dwellings, dependency ratio and socio-economic quintile.

	$logit(P_{survey})$	$logit(P_{ANC})$	Pdensity	Formal	SEQ	DR	MMR	HFB
$logit(P_{survey})$	1	0.71	0.01	-0.36	-0.28	0.29	-0.14	0.09
$logit(P_{ANC})$		1	0.11	-0.41	-0.23	0.16	-0.34	0.14
Pdensity			1	0.05	0.46	-0.47	-0.12	-0.21
Formal				1	0.52	-0.52	-0.07	-0.09
SEQ					1	-0.93	-0.1	-0.18
DR						1	0.14	0.14
MMR							1	-0.01
HFB								1

$logit(P_{survey})$ logit transformation of direct survey domain estimates of HIV prevalence in the general population

$logit(P_{ANC})$ logit transformation of district-level HIV prevalence among pregnant women who obtained antenatal care

Pdensity Population density (km^{-2})

Formal Percentage of residences which are "formal" dwellings

SEQ Socio-economic quintile

DR Dependency ratio

MMR Maternal mortality rate

HFB Percentage of births delivered in health care facilities
